# The Effects of Plasma-Activated Water Treatment on the Growth of Tartary Buckwheat Sprouts

**DOI:** 10.3389/fnut.2022.849615

**Published:** 2022-02-24

**Authors:** Ya Wang, Zihan Nie, Tingjun Ma

**Affiliations:** School of Food Science and Engineering, Beijing University of Agriculture, Beijing, China

**Keywords:** buckwheat, germination, plasma-activated water, nutrients, functional activity, antioxidant activity

## Abstract

The aim was to investigate the effects of buckwheat sprout treated with plasma-activated water (PAW) and their quality, nutrients (protein, amino acids, fat, and carbohydrates), functional active ingredients (total flavonoids, total phenolic acids, γ-gamma aminobutyric acid (GABA), and polysaccharides), and antioxidant activity during germination. PAW had no negative effects on the germination rate, but promoted the stem growth instead, which indicated 1.12-fold higher germination rate compared with the control group. The results of sensory evaluation demonstrated that the obtained sprouts were bright green, shinning, crisp and smooth, with sufficient moisture, and easy to chew. During germination (1–9 days), the water content, amino acids, and reducing sugars of sprouts showed an increasing trend and were basically higher in the PAW group than in the control group, while protein, carbohydrate, and crude fat presented a decreasing trend. The results were that the flavonoid, phenolic acid, γ-GABA, polysaccharides content, and antioxidant activity during germination showed a gradual upward trend but with slight differences, and the antioxidant properties of buckwheat sprouts might be related to the phenolic acid and polysaccharides content. These data show that the PAW treatment on buckwheat sprout have great potential as a dietary source of antioxidant function with health benefits.

## Introduction

Tartary buckwheat (*Fagopyrum tataricum* (L.) Gaertn.) is a dicotyledonous plant that belongs to the *Polygonaceae* family, which is a traditionally edible and medicinal plant (1). Buckwheat is a nutritionally balanced gluten-free crop that has been in the cultivation for 4,000 years and is now grown globally (2). Buckwheat grains are mainly composed of high-quality starch and dietary fiber, protein, lipids, vitamins, amino acids, etc. (3, 4). It is rich in flavonoids (5), phenols (6), proteins and peptides (7), sugar alcohols, D-Chiral inositol (8), polysaccharides (9), steroids, and other bioactive substances, which has a high antioxidant effect by inhibiting the oxidative stress, beneficial to the prevention and treatment of cardiovascular disease, and reduce triglycerides (10), total cholesterol, so it has a very good healthcare, medicinal value (11). However, there is fagopyrin substance in buckwheat, which is a phototoxic naftodianthrone related to hypericin (12). The phototoxic dose of fagopyrin for humans is still unknown (13), some researchers have estimated the recommended daily intake of buckwheat sprouts to be less than 40 g, based on a comparison to hypericin toxicity (14).

Buckwheat has been made into a series of consumer foods, such as bread (15), noodles (16), and honey (17). In addition, buckwheat sprouts are used as functional vegetables which are healthy and unpolluted (18). Buckwheat germination can make phenolic acids, flavonoids, and other active substances content increase significantly, while the antioxidant activity is increased and the taste is also improved (19, 20). Besides, the production process of buckwheat sprouts is simple and convenient with a short reproductive cycle. All this makes the research of buckwheat product worthwhile. The use of some new technologies to promote germination can result in higher quality sprouts, increase active substance content, and promote the sprout growth, such as plasma.

Plasma is an ionized gas consisting of electrons, atoms, ions, radicals, and other molecules (21). The non-thermal (cold) plasma technology is an emerging non-thermal food preservation approach for fresh production. It is investigated as an alternative for the safe and residual-free antimicrobial food treatment, drawing considerable interest in the last decade, and becoming an important factor widely (22). The plasma treatment of water, termed as plasma-activated water (PAW), results in changes of the redox potential, conductivity, and in the formation of reactive oxygen species (ROS) and reactive nitrogen species (RNS) (23). A number of studies have shown that plasma is effective in improving the seed quality, inactivating bacteria (24), and has the potential to be used for the activation of germination and seedling growth (25, 26). It was found that plasma treatments on mung bean seeds could induce significantly more water absorption and lead to a higher rate of germination (27).

The study of buckwheat sprouts becomes very significant. The objective was to investigate the effect of PAW on the germination of buckwheat seeds. Furthermore, the influences of PAW treatment on germination, sensory properties, nutrients, and active substances, and antioxidant properties of buckwheat seeds were also evaluated.

## Materials and Methods

### Materials

Tartary buckwheat seeds (Hefeng No. 1) were cultivated in Datong, Shanxi Province, China, and were acquired from Beijing Green Valley Sprout Co., Ltd. (Beijing, China). Rutin, gallic acid, and γ-Amino butyric acid standard were purchased from Shanghai solarbio Bioscience & Technology Co., Ltd. (Shanghai, China). The analytical grade chemicals, such as Folin-Ciocalteu reagent, gallic acid (GA), sodium carbonate, sodium hypochlorite, potassium dihydrogen phosphate, dipotassium hydrogen phosphate, phosphoric acid, glucose anhydrous, aluminum chloride, potassium acetate, o-phthalaldehyde, copper sulfate, potassium sulfate, sulfuric acid, boric acid, ethanol, ethyl acetate, hydrochloric acid, trichloroacetic acid, sodium acetate, phenol, potassium persulfate, sodium hydroxide, and aluminum chloride were purchased from Shanghai Macklin Biochemical Co., Ltd. (Shanghai, China). In addition, 2,2-diphenyl picryl hydrazyl (DPPH), acetone, and methanol (HPLC grade) were purchased from Shanghai Aladdin Biochemical Technology Co., Ltd. (Shanghai, China). Total antioxidant capacity (T-AOC) kit was purchased from Nanjing Jiancheng Bioengineering Institute (Nanjing, China).

### Preparation of PAW

**Figure 1:**
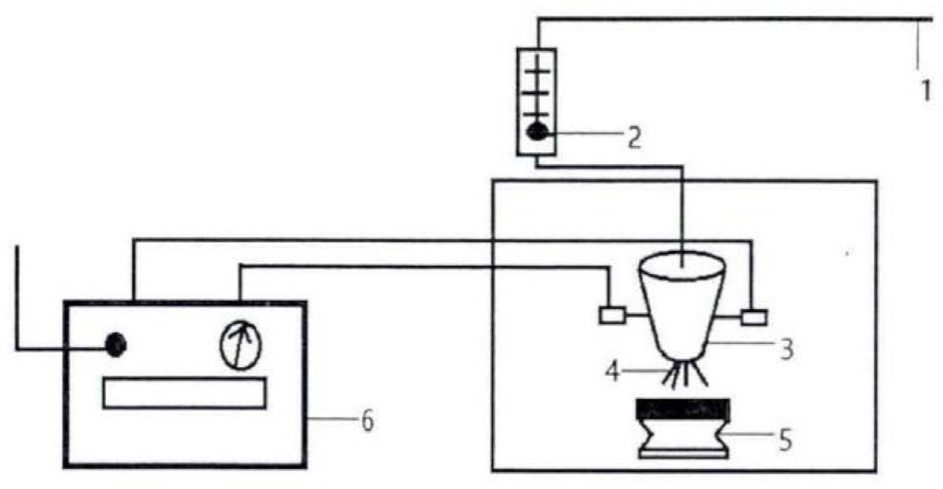
Schematic diagram of experimental set-up for plasma-activated water (PAW) generation. 1. Gas source; 2. Gas flow tester; 3. Reactor; 4. Sliding arc discharge; 5. Sample stage; 6. Power supply.

According to previous studies in the laboratory, PAW was prepared by using a discharge time of 20 min, an action spacing of 0.3 cm, a gas flow rate of 40 L/min, and a discharge power of 450 W (the plasma device is shown in Figure 1). The treated PAW was sprayed on the buckwheat seeds instantly.

### PAW Treatment for Tartary Buckwheat Seeds

Full buckwheat seeds of uniform size and free from mold were selected, washed 3 times, and then activated with water at 65°C. After that, the seeds were soaked with deionized water in a water bath at 30° for 4 h and then removed from water, disinfected with 1% NaClO solution for 15 min, wrapped in a moist breathable soft cloth, and placed in a dark place for germination. Germinated seeds were poured with the appropriate PAW two times a day during germination, until the seeds sprouted.

The germinated seeds were evenly placed in the perforated sprout culture tray and covered with a sheet of paper wetted with PAW, and then placed in the back shade for the germination and growth.

Plasma-activated water was sprayed daily until the buckwheat on the shoot axis reached 1–2 cm, and then the top of the paper was removed. The germinated seeds were grown in a low-light condition (30 μmol/m^2^/s), sprayed with PAW 4 times every day. After 4 days, the sprouts were put into the place with high light (100 μmol/m^2^/s) for growth.

### Buckwheat Sprouts Quality Analysis

#### Germination Rate

The germination rate was determined according to the method proposed by Cao et al. (27). The germinated buckwheat seeds were put into a constant temperature incubator, and a certain temperature to germinate was set for daily observation and recording. The buckwheat was considered to be germinated when the radicle reached 2.0 mm. One hundred grains were taken out randomly after 72 h of germination, and the number of buckwheat sprouts which reached the standard of germination were counted. The germination rate was calculated based on the total 100 buckwheat seeds. Each sample was counted three times and the mean was calculated as the result.


$$\begin{array}{l} Germination\;rate/\%\;=\;\frac{Germinated\;seeds}{Total\;seeds}\times 100\% \end{array}$$

#### Seed Size and Thousand Seed Weight

A total of 100 full seeds were randomly selected, measured their total particle size, and calculated the average single grain size; a total of 1,000 buckwheat seeds were randomly selected and their weight was measured.

#### Root and Stem Lengths

The buds and seedlings on the 10th day were selected and their root and stem lengths were measured by using a ruler. The average value was chosen for data analysis.

#### Sprout Weight

Fifty buckwheat sprouts of the same growth were selected randomly for weight measurement.

#### Moisture Content

Sprouts moisture content was determined gravimetrically, using an oven at 130°C for 3 h. The assay was carried out in triplicate.

#### Sensory Evaluation

Sensory evaluation was determined according to the method proposed by Li et al. (28). The sensory evaluation was made by 40 untrained volunteers. They were staff and students from the Beijing University of Agriculture who were regular sprouts consumers from various socioeconomic backgrounds between the ages of 18 and 50 years. The samples of freshly-made sprouts were evaluated. Panelists were asked to evaluate the brittleness, glossiness, flavor, juiciness, and overall liking using a 9-point hedonic scale ranging from 4 (dislike extremely) to 20 (like extremely). Water was provided to rinse the mouth between evaluations. Finally, panelists were encouraged to write down additional comments.

### Material Treatment

#### Preparation of Buckwheat Sprout Powder

Buckwheat sprouts were treated with a lyophilizer for 24 h, crushed and sieved through 60 mesh, and then stored in a 4°C refrigerator for backup.

#### Crude Extract

Furthermore, 0.5 g of Tartary buckwheat sprout powder was weighed, 35 ml of 70% ethanol was added accurately, and an ultrasonic extraction was applied at 700 W, 40 Hz, and 50°C for 20 min. In addition, the sample was centrifuged at 4,000 r/min for 10 min to obtain the buckwheat crude extract (BCE).

#### Polysaccharides Crude Extract

For this, 20 ml of deionized water was added to 1 g of Tartary buckwheat sprout powder, an ultrasonic extraction was applied for 30 min, 4,000 r/min centrifugation was conducted for 10 min. Then, 2 ml of the deionized water was taken to be dissolved in water and transferred to a 50 ml centrifuge tube. Then, 20 ml of anhydrous ethanol was added and mixed well. After that, the mixture was let stand for 2 h, centrifuged at 4,000 r/min for 10 min. It then followed that the supernatant was discarded and 20 ml of anhydrous ethanol was added. The mixture was then centrifuged at 4,000 r/min for 10 min and washed two times repeatedly. The residue was dissolved in water and transferred to a 10 ml colorimetric tube, diluted to the scale with water, and mixed to obtain the crude extract of buckwheat polysaccharides.

### Nutrients of Buckwheat Sprouts

The amounts of protein, amino acids, carbohydrates, crude fat, and reducing sugar in the Tartary buckwheat flour samples were determined according to the Chinese National Standard method (Standard No. GB 5009.5-2016, GB/T 5009.9-2016, GB5009.6-2016, and GB 5009.7-2016; Standards Press of China, Beijing, China).

### Active Substances of Buckwheat Sprouts

#### Flavonoid Content

The content of total flavonoids was estimated by the method of Gabr et al. (29). Briefly, 1 ml of ethanolic solution AlCl_3_ (0.1 mM) was combined with 1 ml of BCE, after which 1.5 ml of 1 mol/L CH_3_COONa solution, and 1.5 ml 70% ethanol solution were added. The mixture was kept for 1 h at room temperature. The absorbance was measured at 420 nm. The content of total flavonoids was expressed as rutin (RE) [mg RE/g dry weight (DW)].

#### Total Phenolics Content

Total phenolics content of the extract was determined by the method of Park et al. (30) with a slight modification. The BCE (1 ml) was mixed with 6 ml water, 1 ml 5% Folin-Ciocalteu reagent, and 2 ml 5% (w/v) sodium carbonate solution. The mixture reacted for 30 min at 40°C. Absorbance was measured at 765 nm. Total phenolics content was expressed as gallic acid equivalent.

#### Gamma Aminobutyric Acid Content

The freeze-dried powder sample (0.500 g) was mixed with 5 ml 10% trichloroacetic acid, and the samples extracts were shaken on an oscillator for 1 min and then held at 40°C for 2 h to extract the gamma aminobutyric acid (GABA). The extracts were centrifuged at 13,000 ×g for 15 min (31). Then, 0.6 ml of centrifugation supernatant was transferred to standard solution, 0.5 ml of 0.1 mol/L sodium tetraborate solution (pH = 10), 0.4 ml of 6% phenol, and 0.6 ml of sodium hypochlorite (effective chlorine 8%) were added, and 20 min of reaction was applied. Then, 2 ml of 60% ethanol solution was added after it turned blue-green, and the absorbance value was measured at 650 nm.

#### Polysaccharides Content

The total content of polysaccharides was determined using the modified phenol-sulfuric acid method. Briefly, 1 ml of deionized water was added to 1 ml buckwheat polysaccharides crude extract, then 1 ml of 6% phenol solution was added, shaken well, and 5.0 ml of concentrated sulfuric acid was added quickly. After being placed for 5 min, it was heated in a boiling water bath for 15 min, cooled to room temperature, and the absorbance was measured at 490 nm.

### *In vitro* Antioxidant Assays

#### Total Antioxidant

The determination of T-AOC was performed according to the assay kit method, with the absorbance measured at 520 nm.

#### 1-Diphenyl-2-Picrylhydrazyl (DPPH) Radical-Scavenging Activity

The radical scavenging activity of polysaccharides was measured by 1-diphenyl-2-picrylhydrazyl (DPPH) method (32). Briefly, 2 ml of crude extract was mixed with of 0.5 mM DPPH, mixtures were shaken, and left in the dark for 30 min, and absorbance was measured at 517 nm. DPPH free radical scavenging activity (%) was calculated as follows:


$$\begin{array}{l} DPPH\;free\;radical\\ scavenging\;activity/\%=\left[1-\frac{k\left(sample\right)}{k\left(control\right)}\right]\times 100\% \end{array}$$

Where the solution without the sample was the control.

#### 2,2-Azino-Bis (3-Ethylbenzothiazoline-6-Sulfonic Acid) (ABTS) Radical-Scavenging Activity

The 2,2-azino-bis (3-ethylbenzothiazoline-6-sulfonic acid) (ABTS) free radical scavenging activity was determined by the method of Edziri et al. (33). To make ABTS solution, 7 mM ABTS solution, and 2.45 mM potassium persulfate solution were mixed at a ratio of 1:1 and kept overnight at room temperature. The ABTS solution was diluted with water to an absorbance of less than 0.70 at 734 nm before use. Then, 40 μl of BCE was mixed with 1 ml of the diluted ABTS solution. The mixture was shaken for 30 s, and the reaction was carried out for 30 min with protection from light. Absorbance was measured at 734 nm. The ABTS free radical scavenging activity (%) was calculated as follows:


$$\begin{array}{l} ABTS\;free\;radical\\ scavenging\;activity/\%=\left[1-\frac{k\left(sample\right)}{k\left(control\right)}\right]\times 100\% \end{array}$$

Where the solution without the sample was the control.

#### Superoxide Anion Radical Scavenging Ability

The scavenging activity was assessed by the autoxidation of pyragallol in alkaline solution in accordance with the protocol by Mtetwa (34) with slight alterations. Briefly, 200 μl of BCE was added with 5.7 ml of Tris-HCl buffer (50 mM, pH 8.2). Thereafter, 100 μl BCE was added and vortexed for 15 s, subsequently incubated at 25°C for 10 min. The absorbance was measured at 420 nm. The superoxide anion radical scavenging ability was calculated as follows:


$$\begin{array}{l} Superoxide\;anion\;radical\\ scavenging\;ability/\%=\left[1-\frac{k\left(sample\right)-k\left(blank\right)}{k\left(control\right)}\right]\times 100\% \end{array}$$

Where the solution without the sample was the control and the solution without the sample was the blank. Vitamin C was used as the positive control.

### Statistical Analysis

All results are expressed as mean ± SD. Statistical data of three independent replicates were analyzed using the SPSS statistical package 22.0 (SPSS Inc, USA). Data were subjected to two-way ANOVA. Mean comparison was performed using the Duncan's test at a significance level of 0.05 (*p* < 0.05).

## Results

### Growth Indicators of Buckwheat Seeds

As shown in Table 1, the seeds [the weight and size of which were not significantly different (*p* > 0.05)] were treated for germination. The stem length, shoot weight, and germination rate of buckwheat sprouts in the PAW group increased significantly (*p* < 0.05) compared with the control group, and the germination rate reached 93 ± 0.66%, the control group was only 83 ± 0.67%, indicating that the PAW treatment has a positive effect on buckwheat germination, which can promote the growth of roots and facilitate the uptake of water during the seed reproduction.

**Table 1:** Growth index of Tartary buckwheat seeds.

**Group**	**Seed size** **(mm)**	**Weight of 1,000 seeds** **(g)**	**Root length** **(mm)**	**Stem length** **(mm)**	**Sprout weight** **(g)**	**Germination rate** **(%)**
Control	3.87 ± 0.15^a^	27.63 ± 0.17^a^	122.86 ± 0.52^a^	30.58 ± 0.43^a^	1.82 ± 0.80^a^	83 ± 0.67^a^
PAW	3.86 ± 0.20^a^	27.49 ± 0.21^a^	155.52 ± 0.48^b^	45.56 ± 0.68^b^	2.11 ± 0.69^b^	93 ± 0.66^b^

*Values are given as the mean ± SD (n = 3). Different lowercase letters in the same column indicate significant differences (p < 0.05)*.

### Moisture Content

**Figure 2:**
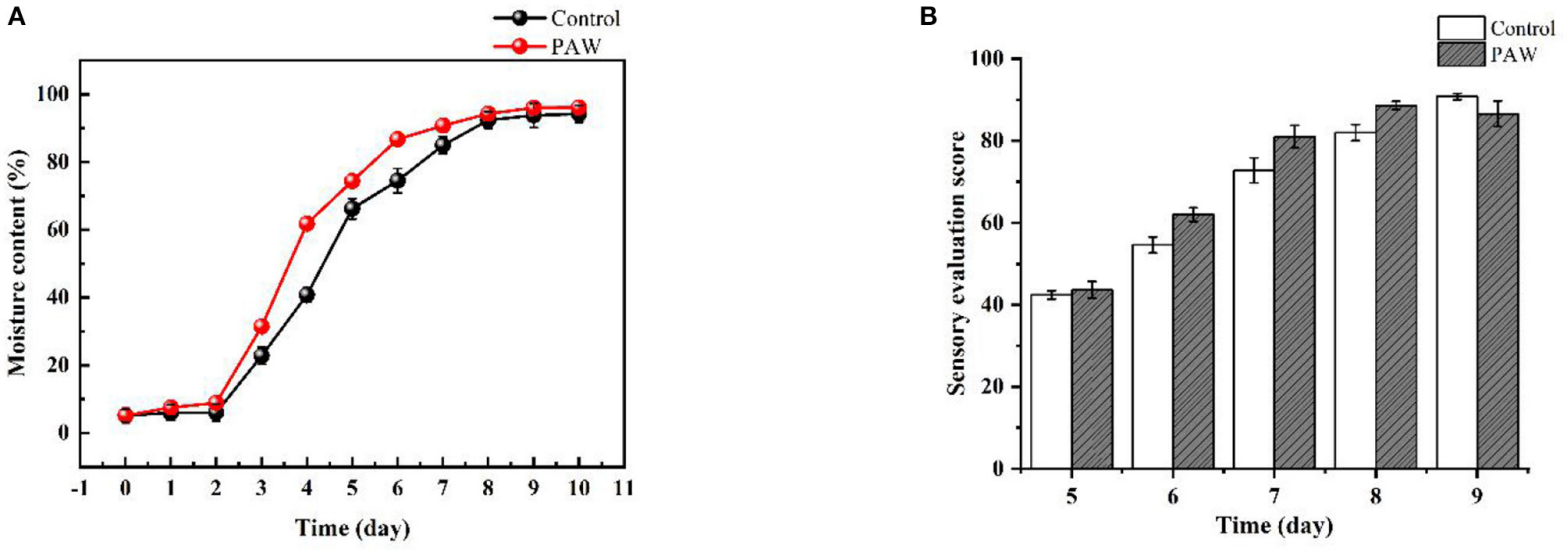
Effects of PAW on moisture content **(A)**, sensory evaluation scores of sprouts in different periods **(B)**.

The effect of PAW treatment on buckwheat sprouts moisture content is shown in Figure 2A. Both the control and PAW groups showed a flat increase followed by a steep increase, but the moisture content of PAW group is higher than that of the control group consistently. The fastest growing period of moisture content of sprouts is from the 2nd to the 6th day. The increase in moisture content for the reproductive process of water absorption has a positive effect, and is also beneficial to the root growth, indicating that the treatment of PAW can promote seed germination and growth of buckwheat sprouts, which means a better hydration of the plants (35).

### Sensory Evaluation

The sensory evaluation scores of sprouts are shown in Figure 2B. Taste panelists could not differentiate between the control and PAW samples (data not shown). There was no significant difference (*p* < 0.05) in brittleness, glossiness, flavor, juiciness, or overall liking of the PAW samples as compared with control. Taste panelists generally agreed that the sprouts were bright green, shinning, crisp and smooth, with sufficient moisture, and easy to chew. The PAW group of sprouts on the 8th day had the highest score and the best overall quality.

### Nutrients of Buckwheat Sprouts

Compared with the seed group, sprouts grown to the 9th day had a significant increase in 16 kinds of amino acids in the control group and a significant increase in 17 kinds of amino acids in the PAW group (18 kinds of amino acids in total). The PAW group had a significant increase of 13 amino acids compared with the control group and the total amount was also higher than the control group (Table 2). Both the PAW and control groups showed an overall balance between a continuous increase in total amino acid content and a continuous decrease in protein content during germination, which was due to the breakdown of protein into small molecule peptides and amino acids during the germination process.

**Table 2:** Amino acid analysis of buckwheat sprouts on the 9th day.

	**Seed (ng/g)**	**Control (ng/g)**	**PAW (ng/g)**
Aspartic Acid	1,516.858 ± 0.1^a^	1,711.999 ± 0.21^b^	1,783.864 ± 0.1^c^
L-Threonine	424.853 ± 0.13^a^	721.111 ± 0.1^b^	739.156 ± 0.21^c^
Serine	830.508 ± 0.2^a^	999.492 ± 0.12^b^	1,046.447 ± 0.21^c^
Glutamic acid	3,777.001 ± 0.15^a^	4,470.853 ± 0.11^b^	4,633.509 ± 0.13^c^
Glycine	1,003.99 ± 0.1^a^	1,112.563 ± 0.13^b^	1,191.033 ± 0.16^c^
Alanine	546.349 ± 0.2^a^	1,066.239 ± 0.20^b^	1,132.401 ± 0.16^c^
L (+)-Cysteine	279.687 ± 0.2^c^	89.208 ± 0.11^b^	82.579 ± 0.13^a^
Valine	987.194 ± 0.2^a^	1,588.843 ± 0.14^b^	1,685.827 ± 0.12^c^
DL-Methionine	88.646 ± 0.2^c^	68.001 ± 0.13^a^	81.785 ± 0.12^b^
L-isoleucine	592.34 ± 0.1^a^	908.701 ± 0.16^b^	964.818 ± 0.15^c^
Leucine	278.23 ± 0.21^a^	352.278 ± 0.12^c^	344.863 ± 0.15^b^
Tyrosine	312.456 ± 0.14^a^	582.69 ± 0.20^b^	623.533 ± 0.13^c^
Phenylalanine	95.9 ± 0.21^a^	122.995 ± 0.13^c^	109.9 ± 0.15^b^
Lysine	1,229.383 ± 0.11^a^	1,914.7 ± 0.13^b^	2,036.545 ± 0.13^c^
Histidine	379.366 ± 0.12^a^	632.662 ± 0.15^b^	678.406 ± 0.15^c^
Arginine	1,907.837 ± 0.11^c^	1,593.887 ± 0.11^a^	1,697.259 ± 0.24^b^
Hydroxyproline	12.56 ± 0.13^a^	539.837 ± 0.22^b^	572.122 ± 0.21^c^
Proline	177.081 ± 0.13^a^	327.739 ± 0.31^b^	346.411 ± 0.21^c^
Total	14,440.239	18,803.798	19,750.458

*Values are given as the mean ± SD (n = 3). Different lowercase letters in the same line indicate significant differences (p < 0.05)*.

**Figure 3:**
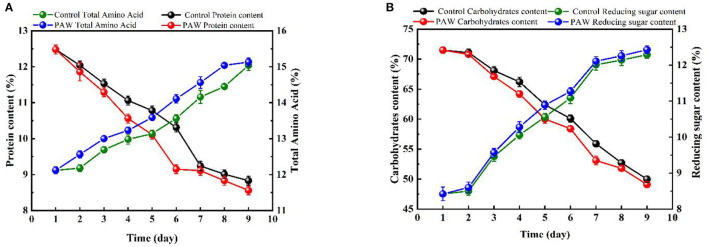
Changes of nutrients during the germination of buckwheat: protein and total amino acid content **(A)**, carbohydrate and reducing sugar **(B)**.

**Figure 4:**
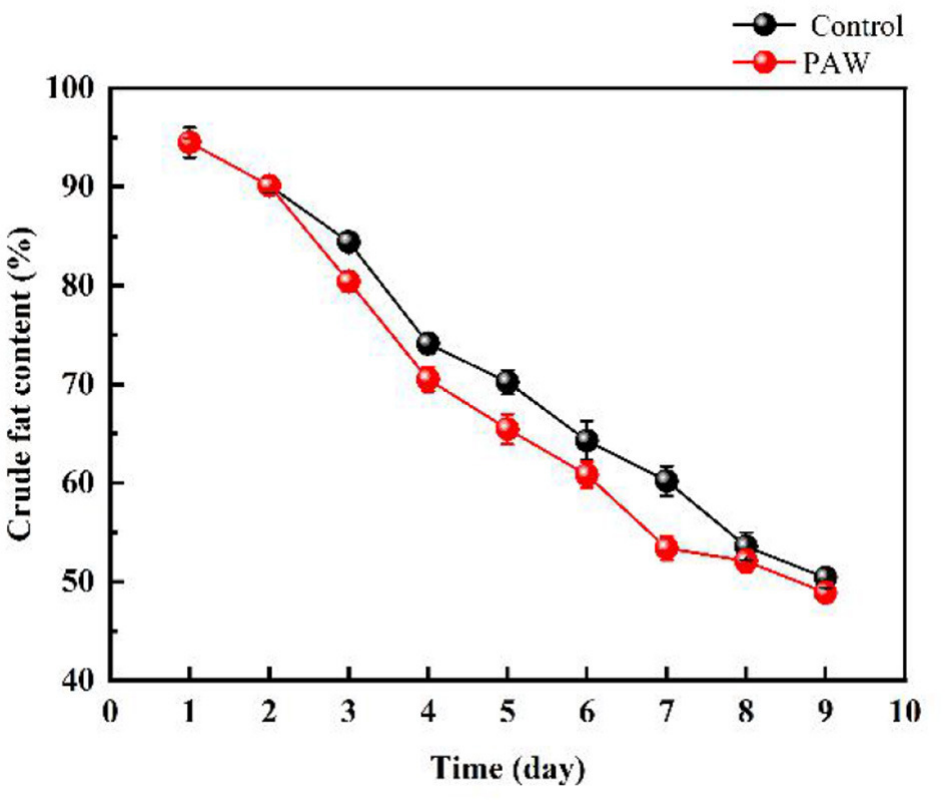
Changes of crude fat during the germination of buckwheat.

As shown in Figure 3A, the protein content of the control group (during 0–7 days) and the PAW group (during 0–6 days) decreased rapidly, in the last 3 days, the protein content decreased at a slower rate because of the impact of proteases during germination. The protein content on 9th day was 8.56 ± 0.121% in the PAW group and 8.83 ± 0.123% in the control group. As shown in Figure 3B, the carbohydrate content of buckwheat germination on the first day basically did not change, which decreased gradually from 1–9 days after germination, during which the starch was decomposed to provide available energy for plant growth. The content of reducing sugar in Tartary buckwheat seeds was 8.42 ± 0.19%, which was as high as 12.29 ± 0.1% on the 9th day after germination. There was no significant difference in the changes of carbohydrates and reducing sugars between the PAW group and the control group. The decrease and increase of carbohydrates and reducing sugars in the PAW group would be more obvious. The crude fat content of buckwheat is shown in Figure 4. During buckwheat seed germination, crude fat declined continuously. Photosynthesis was weak at the beginning of germination and the carbon source was insufficient to be utilized as an energy source, so crude fat was used for energy supplementation and the content started to decrease.

### Active Substances of Buckwheat Sprouts

**Figure 5:**
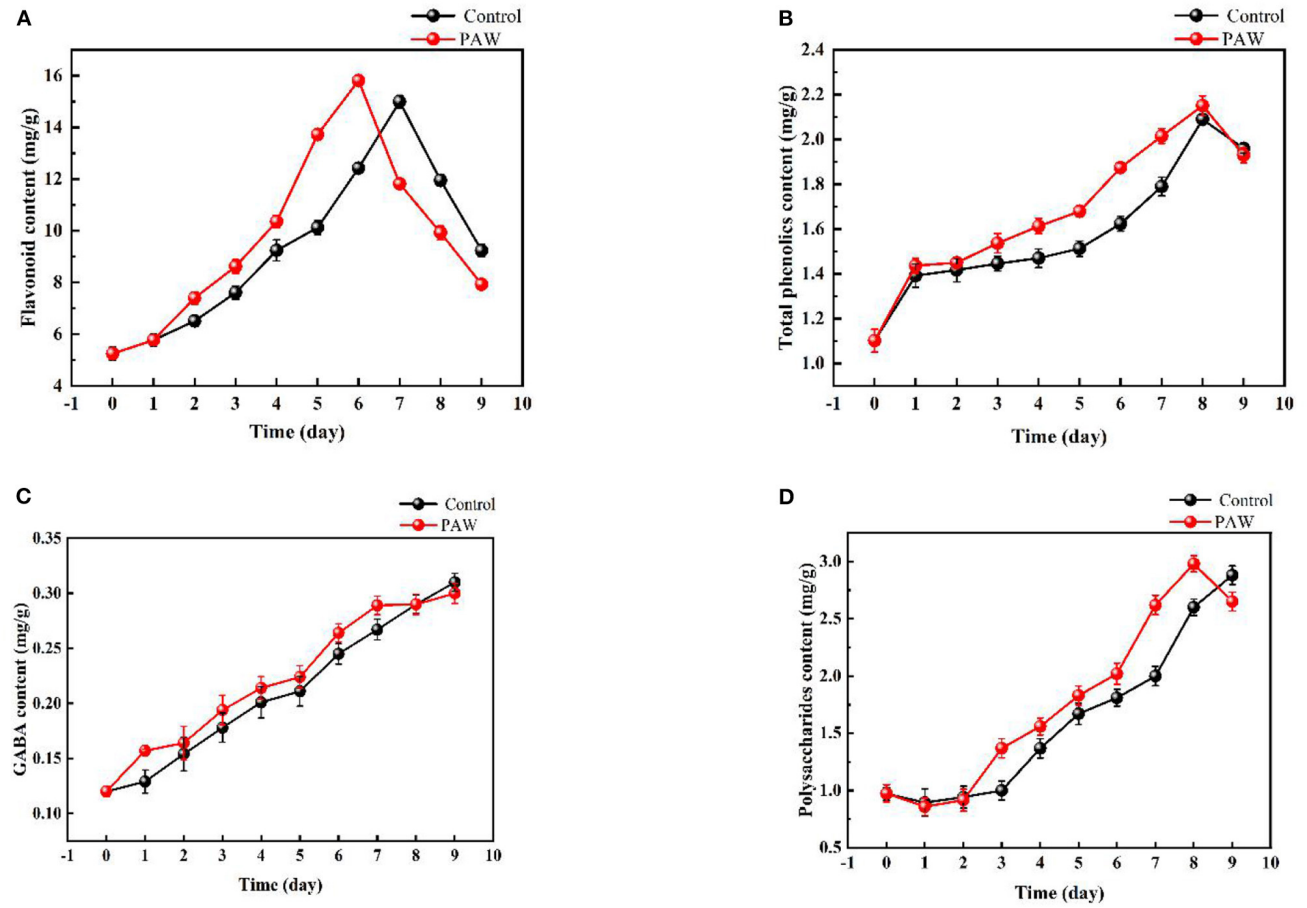
Changes of active substances during the germination of Tartary buckwheat: total flavonoids **(A)**, total phenolic **(B)**, gamma aminobutyric acid (GABA) **(C)**, polysaccharides **(D)**.

As shown in Figure 5A, with the increase of germination time, the content of total flavonoids gradually increased, and the growth rate of flavonoids was relatively fast in the early stage of germination (0–6 days in the PAW group and 0–7 days in the control group). The flavonoid content of Tartary buckwheat seeds was 5.25 ± 0.23 mg/g, and the flavonoid content of dry weight of Tartary buckwheat sprouts in the PAW group reached 15.81 ± 0.21 mg/g on the 6th day of germination, which was three times that of the original seeds and there was a significant difference between the flavonoid content of Tartary buckwheat sprouts and seeds 6 days before germination (*p* < 0.05). The change of total phenolic acid content during the Tartary buckwheat germination is shown in Figure 5B. During 0–9 days of germination, the total phenolic acid content showed an overall gradual increasing trend. On the 8th day of germination, the total phenolic acid content (2.151 ± 0.044 mg/g in the PAW group, and 2.09 ± 0.023 mg/g in the control group) was about 2.1 times that of total phenolic of Tartary buckwheat seed dry weight (1.02 mg/g).

The change of γ-GABA content during the Tartary buckwheat germination is shown in Figure 5C. With the increase of germination time, γ- GABA content raised as well. The maximum amount was reached on the 9th day of germination (0.30 ± 0.009 mg/g in the PAW group and 0.31 ± 0.008 mg/g in the control group), which was nearly three times that of seeds. With the increase of germination time, the content of polysaccharides also increased (Figure 5D). The maximum polysaccharides content of the control group was 2.88 ± 0.082 mg/g on the 9th day of germination, and that of PAW group was 2.98 ± 0.070 mg/g on the 8th day, which was 2.7 times that of seeds. Then, the polysaccharides content of the PAW group started to decrease, which was consistent with the trend of flavonoid content in the PAW group.

### *In vitro* Antioxidant Assays

**Figure 6:**
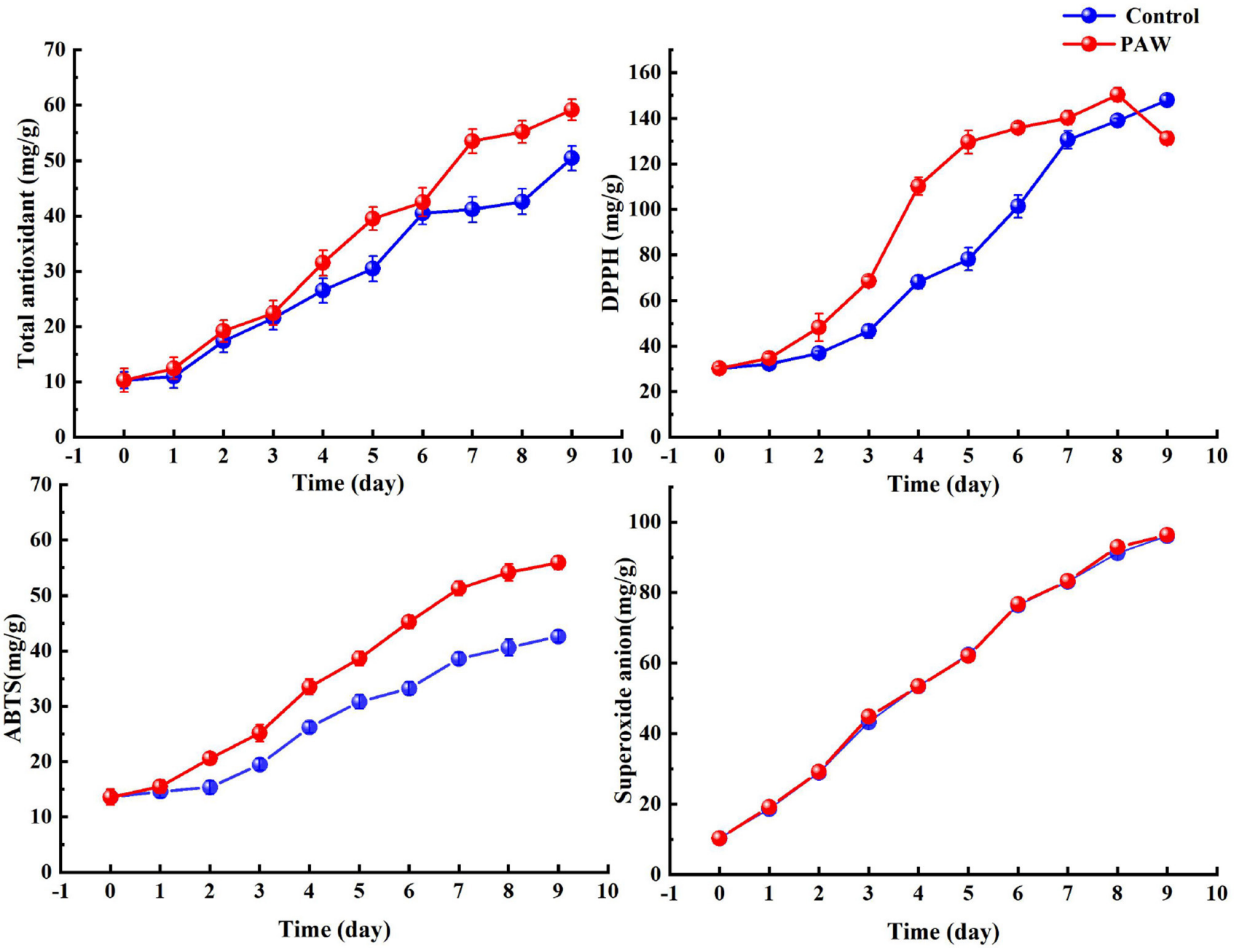
Changes of the antioxidant activity during the germination of Tartary buckwheat.

The changes in antioxidant values during germination in the control and PAW groups are shown in Figure 6, and overall both increased with time, because for both the control and PAW groups, the germination significantly increased the antioxidant activity. The total antioxidant capacity was not significantly different between the control and PAW groups from 0–6 days. However, the PAW group increased rapidly on the 7th day, reaching a maximum value of 59 ± 1.9 mg/g on the 9th day. The DPPH scavenging capacity of PAW group reached a maximum (150 ± 3.1 mg/g) at the 8th day of germination, which was 5 times higher than that of buckwheat seeds, with the same trend of total phenolic acids and polysaccharides.

The changing trend of ABTS radical scavenging ability in the control group and the PAW group was similar. The scavenging rate of sprouts cultivated on 9th day reached 55.95 ± 1.2 mg/g, which was 13.36 mg/g higher than that of control and 4.1 times higher than that of seeds. The superoxide anion radical scavenging capacity also increased with germination time, reaching the maximum amount in both groups at the 9th day of germination (96.4 ± 0.79 mg/g in the PAW group and 96.1 ± 0.77 mg/g in the control group), but there was no significant change in both during the germination process.

## Discussion

The root length, stem length, sprout weight, and germination rate of PAW-treated sprouts were higher than those of the control group, this may be attributed to the fact that the cold plasma treatment increased the activity of metabolic enzymes associated, and reduced the impact of oxidative reactions caused by ROS in plant cells, thus improving root vigor (36, 37). Filatova's research showed that the plasma treatment causes cracks on the seed surface and this “erosive” effect can increase the total surface energy and hydrophilicity, thus promoting the root growth (38). It also has been reported that PAW could significantly stimulate mung bean seeds germination and growth, which might be related to the active components in PAW (39). Hence, we can observe that the PAW treatment has a promotional effect on the growth of buckwheat sprouts. The results of the sensory evaluation revealed that taste panelists were unable to distinguish the taste of PAW and control samples and they liked the PAW samples and the controls equally. Thus, PAW did not affect the overall acceptability of the sprouts. During germination, the reducing sugar content of sprouts increased, which might improve the taste of sprouts.

In the process of buckwheat germination, protein, crude fat, and carbohydrate content follow a downtrend, while amino acids and reducing sugars are the opposite. Protein content decreases rapidly in the early stages of seed germination, which is consumed as the most important source of energy during buckwheat germination, germination also leads to increase the *in vitro* digestibility of proteins (40). As buckwheat contains some amino acids, these decompositions are dispersed to the germ, at which time the germ is activated to synthesize new proteins, so the rate of change in protein content slows down in the later stages. Ishikawa (41) found that the proteolysis reaction and metabolic reactions of amino acids might coexist in sprouts, which explains the increased amino acid content. Carbohydrates are used as the main source of energy for plants, which includes numerous components, such as starch and complex polysaccharides. Amylase is able to breakdown starch into monosaccharides and oligosaccharides. The taste and digestibility of buckwheat could be improved because of the increase of reducing sugar. In our study, the trend in carbohydrate content was gradually decreasing, while some studies have shown that the flavonoids in buckwheat (rutin, quercetin, and kaempferol) inhibit the activity of α-amylase, which leads to a slower rate of starch breakdown (5). Therefore, whether the increase in flavonoid content of buckwheat sprouts obtained by the PAW treatment decreases the rate of carbohydrate decomposition remains to be studied subsequently.

A growing amount of evidence for the role of PAW in the promotion of sprout germination. The experimental results indicated that PAW has been proved to be effective in promoting the growth of buckwheat sprouts, but also in increasing the content of bioactive compounds and enhancing the antioxidant capacity. Scholars have found that the accumulation of flavonoids might be closely linked to the increase in FtFLS2 (one flavonol synthetase isoform gene) expression (20). Their study demonstrated that the flavonoid content showed an increasing trend followed by a decreasing trend during germination. The increases in total phenolic acid might be due to the increase in phenylalanine ammonialyase (PAL) catalyst during germination (42). GABA is a central nervous system (CNS) transmitter that exhibits hypolipidemic and hypocholesterolemic effects. The persistent increase in GABA content suggests that buckwheat sprouts may have the potential as a therapeutic food for the dietary intervention in patients with hyperlipidemia. Earlier studies have shown that the DPPH radical-scavenging activity may be attributed to the fact that the amount of flavonoid ingredients and phenolic acid significantly increased during common buckwheat germination (43), the DPPH assay were strongly correlated to the content of total phenolic acids and proanthocyanidins level in the cotyledons (44). The results of ABTS radical-scavenging activity were essentially the same as those obtained by Živković Andrej's study (45), compared with phenolic content, the antioxidant activity (DPPH and ABTS) measures showed similar trends during the course of germination. However, the changes in flavonoids and phenolic acid monomers and the relevance to the antioxidant properties of sprouts were not studied, so in future studies, the effect of PAW on antioxidant activity in buckwheat sprouts can be considered by targeted metabolomics and analyze the intrinsic mechanism, and improve the study of changes in relevant enzymes in parallel with a view to provide a basis for their nutrient changes.

## Conclusions

The data obtained showed that the PAW treatment had no negative impact on buckwheat germination, but instead promoted the growth of sprouts, improved the germination rate, increased the content of active substance, and enhanced their antioxidant activity. These results indicated that PAW could significantly stimulate buckwheat seeds germination and growth, which might be considered as a promising technology to improve the seeds germination and seedling growth. The PAW treatment provides ideas for the preparation of functional foods made from buckwheat, but the mechanisms involved still need to be studied.

## Data Availability Statement

The original contributions presented in the study are included in the article/supplementary material, further inquiries can be directed to the corresponding author/s.

## Author Contributions

YW, ZN, and TM: conceptualization, methodology, software, validation, formal analysis, investigation, resources, data curation, and visualization. YW and ZN: writing—original draft preparation. YW and TM: writing—review and editing. TM: supervision. All authors contributed to the article and approved the submitted version.

## Conflict of Interest

The authors declare that the research was conducted in the absence of any commercial or financial relationships that could be construed as a potential conflict of interest.

## Publisher's Note

All claims expressed in this article are solely those of the authors and do not necessarily represent those of their affiliated organizations, or those of the publisher, the editors and the reviewers. Any product that may be evaluated in this article, or claim that may be made by its manufacturer, is not guaranteed or endorsed by the publisher.
